# Differences in Metabolic Characteristics of Rhizosphere Fungal Community of Typical Arboreal, Shrubby and Herbaceous Species in Oasis of Arid Region

**DOI:** 10.3390/jof10080565

**Published:** 2024-08-10

**Authors:** Yunxiang Tan, Yunhang Lv, Mengyu Xv, Laiye Qu, Wenjuan Wang

**Affiliations:** 1 School of Ecology, Hainan University, Haikou 570228, China; bioecotan@163.com (Y.T.); lvyh@hainanu.edu.cn (Y.L.); xumy@hainanu.edu.cn (M.X.); 2 Research Center for Eco-Environmental Sciences, Chinese Academy of Sciences, Beijing 100085, China; lyqu@rcees.ac.cn

**Keywords:** arid oasis, life form, rhizosphere fungal community, metabolic characteristics, soil property

## Abstract

*Populus euphratica*, *Tamarix ramosissima*, and *Sophora alopecuroides* are, respectively, typical arboreal, shrubby, and herbaceous species in oases of arid regions. It is important to study the difference in metabolic characteristics of the rhizosphere fungal community of these plant species and their relationships with soil factors for the preservation of delicate arid oasis ecosystems with future environmental changes. In this study, we, respectively, collected 18 rhizosphere soil samples of *P. euphratica*, *T*. *ramosissima*, and *S. alopecuroides* to explore the difference in rhizosphere fungal metabolic characteristics of different plant life forms and their underlying driving factors. The results showed that (1) soil physicochemical properties (including soil water content, pH, etc.) were significantly different among different plant species (*p* < 0.05). (2) Rhizosphere fungal metabolic characteristics were significantly different between *S. alopecuroides* and *T. ramosissima* (ANOSIM, *p* < 0.05), which was mainly caused by the different utilization of carboxylic carbon. (3) The RDA showed that the main driving factors of the variations in rhizosphere fungal metabolic characteristics were different among different plant species. The main explanatory variables of the variations in the metabolic characteristics of the rhizosphere fungal community were carbon to nitrogen ratio (23%) and available potassium (17.4%) for *P. euphratica*, while soil organic carbon (23.1%), pH (8.6%), and total nitrogen (8.2%) for *T. ramosissima*, and soil clay content (36.6%) and soil organic carbon (12.6%) for *S. alopecuroides*. In conclusion, the variations in rhizosphere fungal metabolic characteristics in arid oases are dominantly affected by soil factors rather than plant life forms.

## 1. Introduction

As an important part of soil microorganisms, soil fungi participate in nutrient cycling, form symbioses with plants, and play key roles in promoting plant growth and regulating ecosystem processes [1]. The characteristics of fungal carbon metabolism serve as important indicators of fungal function [2], which not only reveals the use of carbon sources by fungi for growth and reproduction but also reflects the trophic role and ecological function of fungi in ecosystems [3,4]. Biolog-FF Microplate is a straightforward and rapid method to measure the diversity of fungal metabolism in the environment based on fungal metabolic response patterns induced by different substrates [5]. Previous studies showed that fungal growth and reproduction and their diversity were influenced by plant, soil, and other factors [6,7]. Therefore, the metabolism of the rhizosphere fungal community could be varied among plant life forms and their relationships with soil properties could be different among plant life forms as well.

Arid zones are ecologically fragile, and oases are the areas with the greatest concentration of energy and material in arid zones [8]. Rhizosphere fungi play an important role in promoting growth and adaptation to harsh environments of oasis plants. It is of great significance to explore the metabolic diversity of the rhizosphere fungal communities of different plants for vegetation restoration and conservation in arid zones. The Ejina oasis is a typical oasis ecosystem in the arid zone of China, which is an important ecological defense line in northwestern China [9,10]. Oases are dominated by open forest landscapes such as riparian forests and shrub forests with relatively simple species composition and structure. *Populus euphratica*, *Tamarix ramosissima*, and *Sophora alopecuroides* are, respectively, dominant in the arboreal, shrub, and herb layers, while they have different adaptation characteristics to the arid zone environment [11]. *P. euphratica* is a typical riparian forest species, with salinity and drought resistance, windbreak, and sand-fixing characteristics, and plays an important role in maintaining the balance of the ecosystem and protecting biodiversity [12]. *T. ramosissima* is a key species in the desert ecosystem, which is usually regarded as one kind of saline- and drought-tolerant shrub, whose unique ecological adaptability enables it to prevent wind erosion, fix sand dunes, and improve saline soils. *S. alopecuroides* is a kind of perennial herbaceous plant that belongs to the family *Fabaceae*, with a strong nitrogen-fixing ability and good drought and wind–sand resistance properties [13], and is widely distributed in arid deserts and grassland margins [14]. Currently, studies on oasis plants in the arid zone mainly focus on the spatial distribution patterns of vegetation [15,16], species-environment relationships [17,18,19], and soil carbon and nitrogen characteristics under typical plant communities [20]. However, it is still not clear whether the carbon metabolic characteristics of the rhizosphere fungi are different among different plant life forms in arid oases.

Therefore, we collected rhizosphere soil samples from different plant life forms (*P. euphratica*, *T. ramosissima,* and *S. alopecuroides*) to explore the differences in the carbon metabolism of rhizosphere fungal communities among different plant life forms in arid oasis by using Biolog-FF Microplate [21]. Then, combined with the soil physicochemical properties, we explored the main soil factors affecting the carbon metabolic characteristics of rhizosphere fungal communities under each plant species. Our findings may provide new knowledge in guiding the conservation and restoration of oasis vegetation in the arid zone.

## 2. Study Area and Research Methodology

### 2.1. Overview of the Study Area

The study area is located in the Ejina Oasis, downstream of the Heihe River in the Inner Mongolia Autonomous Region (100°55′~101°19′ E, 41°47′~42°20′ N), with a flat topography and an elevation ranging from 900 to 1100 m. The climate in this area is arid, featuring sparse precipitation, with average annual precipitation lower than 40 mm and annual minimum precipitation of up to 7 mm; both evapotranspiration and evaporation are high, with average annual values of about 3249 mm, and a peak annual evaporation of up to 4000 mm; the air’s relative humidity is below 35%, the average annual temperature is 9.5 °C, and the average annual wind speed is 4.2 m/s [22].

### 2.2. Experimental Sample Collection

In three 100 m × 100 m long term monitoring plots with *P. euphratica* as a building species in Ejina National Nature Reserve, Alashan, Inner Mongolia, we first randomly selected six individuals of *P. euphratica*, *T. ramosissima*, and *S. alopecuroides*, respectively, in each plot during the growing season (June to August). Then, rhizosphere soil samples were collected in a range of 0.5 m with plant bases in four directions (south, east, north, and west) for each selected plant. For each sampling point, the plant roots in the 0–20 cm soil layer were excavated, and then the rhizosphere soil was collected by shaking these roots. The four subsampled soils from four directions were mixed to form a composite soil sample of each selected plant individual and then were transported to the laboratory in an ice box. In total, 54 rhizosphere soil samples under *P. euphratica*, *T. ramosissima*, and *S. alopecuroides* were collected in this study. After removing and discarding roots and stones with a 2 mm mesh screen, these rhizosphere soil samples were stored and further used for Community Level Physiological Profiling (CLPP) and the determination of soil physicochemical properties.

### 2.3. Indicator Measurement

The CLPP of the rhizosphere fungal community was determined by the Biolog-FF Microplate method [23]. In brief, fresh rhizosphere soil samples equivalent to 10 g of dried soil were weighed and transferred to a sterile triangular flask. After adding 90 mL of sterile water with 0.85% NaCl, the triangular flask was sealed and shaken at 200 r/min for 30 min, then placed in an ice bath for 2 min. Then, 5 mL of the supernatant was transferred to another 100 mL sterilized triangular flask, to which 45 mL of sterile water was added. This dilution process was repeated three times to achieve a 1:1000 extraction solution. Subsequently, 150 μL of the final extraction solution was added to each well of the Biolog-FF Microplate by micropipette and incubated at a constant temperature of 25 ± 1 °C for 240 h. During this period, the absorption values of each well at 490 nm were measured by a precision Biolog-FF Microplate reader every 24 h.

Soil water content (WC) was determined by the drying method. Soil pH was determined by a digital pH meter (PHS-3E, REX, Shanghai, China; 1:5 *w*/*v*). Soil electrical conductivity (EC) was measured by a conductivity meter (DDS-307A, REX, Shanghai, China). Soil total carbon (TC) and total nitrogen (TN) content were determined by an elemental analyzer. Soil total phosphorus (P), total potassium (K), total sodium (Na), and total calcium (Ca) content were determined by ICP-OES after microwave digestion [24]. Available nitrogen (AN) content was determined by the alkaline hydrolysis diffusion method [25]. Available phosphorus (AP) content was determined by an ultraviolet spectrophotometer [26,27]. Available potassium (AK) content was determined by the ammonium acetate leaching method [28]. Soil organic carbon (SOC) was determined by the K_2_Cr_2_O_7_ oxidation method [26]. Finally, soil particle composition was analyzed by a Mastersizer 2000 laser particle size analyzer (Malvern Instruments, Malvern, UK), which divided the soil into clay (<2 μm), silt (2–50 μm), and sand (>50 μm).

### 2.4. Data Analysis

The overall metabolic intensity of the rhizosphere fungal community was expressed as Average Well Color Development (AWCD), which was calculated based on the absorption values of each well in the Microplate. The calculation formula is as follows:

$$AWCD=\sum (C_{i}-R)/N$$

where $C_{i}$ is the absorption value of the carbon source wells; R is the absorption value of the control well; N is the number of carbon sources (95). The relative absorption values of each carbon source well: $OD=C_{i}-R$.

Soil physicochemical properties and the carbon metabolic intensity of the rhizosphere fungal community of the different plant life forms were analyzed by the One-way Analysis of Variance (one-way ANOVA) using the SPSS software (version 27), and multiple comparisons were performed using the Least Significant Difference (LSD) method. The variations in 95 carbon source metabolism by rhizosphere fungal communities among different plant life forms were analyzed by the ANOSIM method based on the Bray–Curtis distance, which was performed with the vegan package (version 2.5-2) in R-4.3.2. Redundancy analysis (RDA) was used to determine the effects of soil physicochemical properties on the metabolic characteristics of rhizosphere fungal communities; before that, soil properties were screened based on the variance inflation factor (VIF < 30) and forward selection. Finally, the relative explanation of the retained soil factors in the final RDA model was calculated with the rdacca.hp function in the rdacca.hp package (version 1.0-8) [29,30]. The RDA results were presented using the Canoco software (version 5.0).

## 3. Results and Analysis

### 3.1. Soil Physicochemical Properties among Different Plant Life Forms 

The physicochemical properties of the rhizosphere soils were significantly different among these three plant life forms (Table 1). The rhizosphere soil pH and carbon to nitrogen ratio (C/N) of *P. euphratica* were significantly lower than those of *T. ramosissima* and *S. alopecuroides* (*p* < 0.05). The total nitrogen content of *P. euphratica* was significantly higher than that of *T. ramosissima* and *S. alopecuroides*. In addition, the total carbon, soil organic carbon, and available nitrogen content of *P. euphratica* were significantly higher than that of *T. ramosissima* (*p* < 0.05), while the total sodium content of *P. euphratica* was significantly lower than that of *T. ramosissima* (*p* < 0.05). The soil sand content of *P. euphratica* was significantly higher than that of *S. alopecuroides* (*p* < 0.05), while the soil clay content and total calcium content of *P. euphratica* were significantly lower than those of *S. alopecuroides* (*p* < 0.05). The soil water content of *T. ramosissima* was significantly lower than that of *P. euphratica* and *S. alopecuroides* (*p* < 0.05), whereas the total phosphorus content of *T. ramosissima* was significantly higher than that of *P. euphratica* and *S. alopecuroides* (*p* < 0.05).

### 3.2. Carbon Metabolic Characteristics of Rhizosphere Fungal Communities among Different Plant Life Forms

**Figure 1:**
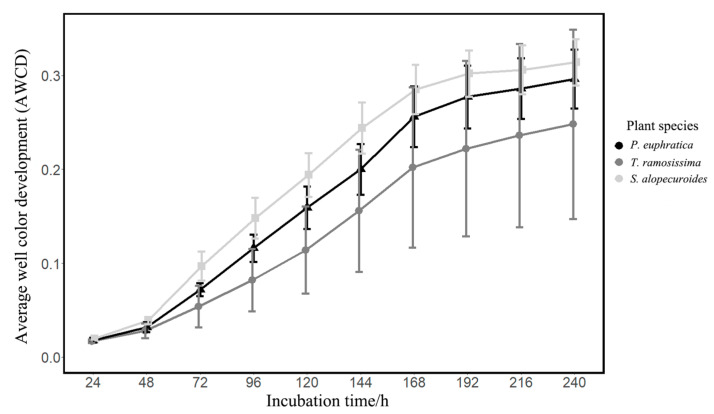
The AWCD values of the different plant life forms during the Biolog-FF Microplate incubation.

The AWCD values reflect the overall intensity of carbon source utilization by the rhizosphere fungal community. Our results showed that the AWCD values of these three plants all gradually increased with the extension of incubation time (Figure 1). At the early incubation stage (0~48 h), the curves were relatively flat and the AWCD values were relatively small (Figure 1), indicating that the intensity of carbon source utilization by the rhizosphere fungal community was relatively lower at the early period. Next, the curve became steeper and the AWCD values increased rapidly until the 168 h incubation point, then the trend turned smooth and the AWCD values changed little (Figure 1). These results suggested that the carbon sources were fully utilized by the fungal community at the 168 h incubation point and the intensity of carbon source utilization would decrease in the next period. Therefore, the subsequent analysis would be based on the data of the carbon metabolic characteristics of the rhizosphere fungal community at the 168 h incubation point.

**Figure 2:**
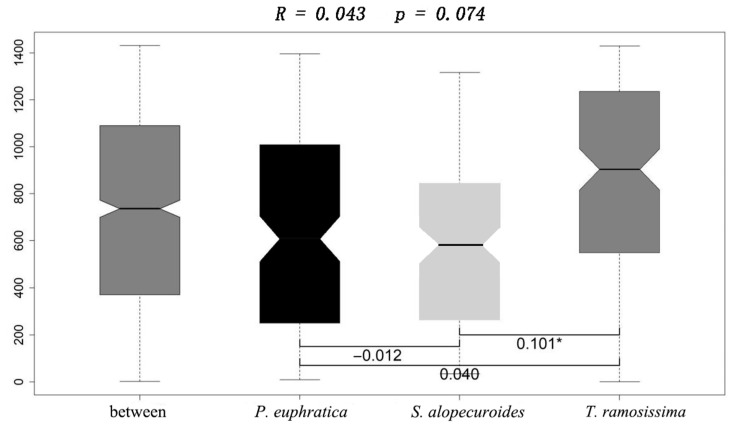
Differences in metabolic characteristics of rhizosphere fungal community among different plant life forms. Notes: * denotes *p* < 0.05.

**Figure 3:**
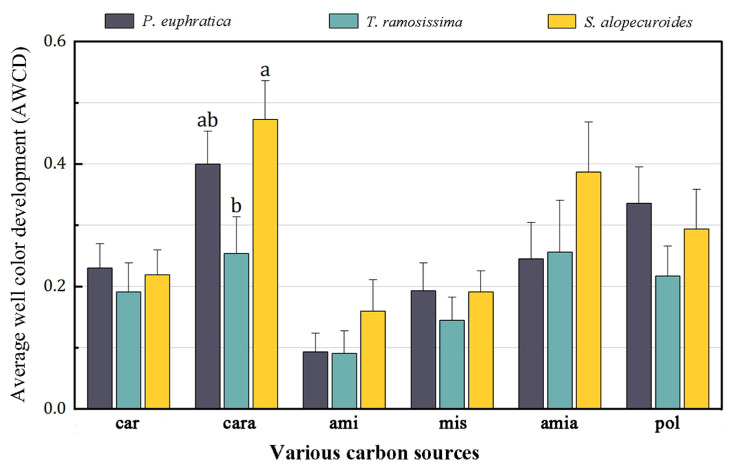
Utilization intensities of the six kinds of carbon sources by the rhizosphere fungal community of the different plant life forms. Note: different letters indicate significant differences at the 0.05 level.

The result of ANOSIM revealed that the metabolic characteristics of the 95 carbon sources significantly differed between *T. ramosissima* and *S. alopecuroides* (R = 0.101, *p* < 0.05; Figure 2). Then, we classified these 95 carbon sources into six groups according to the nature of their chemical groups, namely carbohydrates (car), carboxylic acids (cara), amines (ami), miscellaneous (mis), amino acids (amia), and polymers (pol) [31,32] to further compared the differences in utilization intensity of these six kinds of carbon sources by the rhizosphere fungal community among these three plants. Among these carbon sources, we only found significantly different utilization of carboxylic acid sources among different plants, with a lower utilization intensity of carboxylic acid sources by the rhizosphere fungal community of *T. ramosissima* than that of *S. alopecuroides* (Figure 3).

### 3.3. Relationships between Carbon Metabolic Characteristics of Rhizosphere Fungal Communities and Soil Properties among Different Plant Life Forms

**Figure 4:**
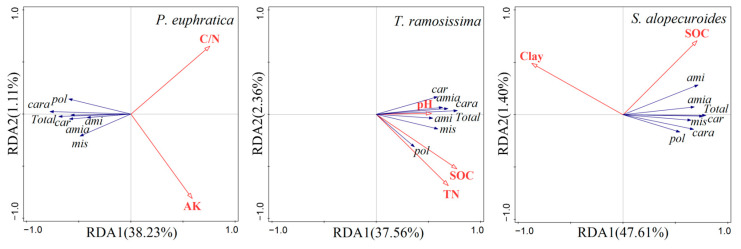
Redundancy analysis of carbon metabolic characteristics of rhizosphere fungal community of different plants.

The results of RDA showed that RDA1 could reflect 38.23% variations in the carbon metabolic characteristics by the rhizosphere fungal communities of *P. euphratica*; moreover, it was positively correlated with C/N and AK (Figure 4). Furthermore, C/N and AK could explain 23% and 17.4% variations in the carbon metabolic characteristics by the rhizosphere fungal communities of *P. euphratica*, respectively (Table 2). For the rhizosphere fungal community of *T. ramosissima*, RDA1 could reflect 37.56% variations in the carbon metabolic characteristics, which was positively correlated with SOC, pH, and TN (Figure 4). And the explanations of SOC, pH, and TN for the variations in the carbon metabolic characteristics were 23.1%, 8.6%, and 8.2%, respectively (Table 2). Finally, RDA1 could reflect 47.61% variations in the carbon metabolic characteristics of the rhizosphere fungal communities of *S. alopecuroides*, which was positively correlated with SOC whereas negatively correlated with clay (Figure 4). The clay and SOC could explain 36.6% and 12.6% variations in the carbon metabolic characteristics of the rhizosphere fungal communities, respectively (Table 2).

## 4. Discussion

### 4.1. Differences in Soil Properties of Different Plant Life Forms

This study showed that the soil physicochemical properties were significantly different among the different plant life forms, such as the soil water content of *P. euphratica* and *S. alopecuroides* was significantly higher than that of *T. ramosissima*, whereas *T. ramosissima* had a higher total sodium content than that of *P. euphratica* and a higher total phosphorus content than that of *P. euphratica* and *S. alopecuroides* (Table 1). This may stem from the fact that different plant life forms in arid oases had different adaptation and modification strategies to the soil environment [33]. Compared with *P. euphratica*, *T. ramosissima* has a stronger competitive ability for groundwater [34] because it is one kind of deep-rooted submersible plant [35,36,37] and has a root system with the ability to absorb water from water-unsaturated soil [38,39]. In addition, *T. ramosissima* is a water-intensive C3 plant [40]. In the dry season, it mainly releases water by increasing stomatal conductance to reduce its own temperature to resist high-temperature stress [41,42], which would lead to low rhizosphere soil water content. As a deep-rooted plant, *P. euphratica* has a strong ability of water redistribution [43], which can transfer deep water to the surface layer of soil to meet the water demand of shallow-rooted herbaceous plants such as *S. alopecuroides* through hydraulic lifting [44]. During the regulation process of soil water content, *P. euphratica* could promote the vertical exchange of salt ions as well so as to avoid the unidirectional movement of salt ions from the deep soil layer to the surface soil layer by evapotranspiration. This process may be the reason for the lower soil pH of *P. euphratica* than that of *T. ramosissima* and *S. alopecuroides*. In addition, *T. ramosissima* has a strong ability to secrete and return salt to the soil surface layer through its dead branches and leaves [45], which causes the “salt island effect” of *T. ramosissima*’s surface soils [46], resulting in a higher total sodium and total phosphorus contents in the rhizosphere soil of *T. ramosissima* than that of *P. euphratica* and *S. alopecuroides*. In addition, calcium plays an important role in maintaining the physiological homeostasis and stability of plant leaf cells, and *P. euphratica* enriches calcium ions in its leaves to mitigate the damage caused by extreme environmental stresses [47]. This unique mechanism makes *P. euphratica* take up calcium ions from the soil to meet its own needs, further resulting in a lower calcium ion content of the *P. euphratica* rhizosphere soil than that of *S. alopecuroides*.

Plant residues are important sources of soil organic carbon, and microorganisms are able to decompose and mineralize plant residues to increase carbon and nutrients in the soil [48]. Our results showed that *P. euphratica* has a higher carbon and nitrogen content in the rhizosphere soil than the other two plants (Table 1), which may be related to the fact that *P. euphratica* is a typical deciduous arbor in desert areas and has a higher output per unit of litter than grassy shrubland vegetation [49]. Previous studies have demonstrated that the level of carbon to nitrogen ratio reflects the growth characteristics and the balance between the competitive and defensive strategies of plants [50]. *P. euphratica* is one kind of tall plant species with a high light compensation point [51,52]. The lower carbon to nitrogen ratio of *P. euphratica* compared with that of *S. alopecuroides* and *T. ramosissima* reflected that *P. euphratica* adopts a competitive strategy while the other species choose a defensive strategy to resist extreme environments. Such different growth strategies might be a reason for the different carbon to nitrogen ratios between *P. euphratica* and the other species. Meanwhile, a previous study has demonstrated that soil organic carbon accumulation influences phosphorus and nitrogen accumulation to a certain extent [53]. In addition, the particle composition of soil can significantly affect the decomposition of plant residues [54,55]. Our result showed that the *P. euphratica* rhizosphere soils had a relatively homogeneous content of sand and silt (Table 1), which could better maintain aeration and water-holding capacity and further promote plant residues decomposition and increase the content of carbon and nitrogen in the soil surface layer.

### 4.2. Differences in Carbon Metabolic Characteristics of Rhizosphere Fungal Community among Different Plant Life Forms

There was no significant difference in the utilization of five kinds of carbon sources, including carbohydrate, amine, others, amino acid, and polymer, by the rhizosphere fungal community among the different plant life forms in arid oases (Figure 3), which may be related to the fact that all of these plants lived in the same water and salt stresses [56]. Despite the microenvironments of the rhizosphere soils of *P. euphratica*, *T. ramosissima*, and *S. alopecuroides* being significantly different (Table 1), all the biomes, including fungi, have to firstly subject to the selection of extreme environments, such as water and salt stresses, and only tolerant fungal species can survive and reproduce [57], so the similar composition of the fungal community in the rhizosphere soils from the different plant life forms retained and exhibited similar carbon metabolic characteristics.

Nevertheless, we still found significant differences in the metabolic intensity of the rhizosphere fungal communities between *S. alopecuroides* and *T. ramosissima* (Figure 2), which mainly resulted from the utilization of carboxylic acid carbon sources by their rhizosphere soil fungi (Figure 3). Carboxylic acids are the main components of plant root secretions and one of the major carbon sources for plant rhizosphere microorganisms [58], as well as a sensitive carbon source for changes in fungal communities [59,60]. Different plants have different types and properties of root secretions, as well as differences in their rhizosphere microenvironments (Table 1), all of which would affect the community composition of rhizosphere fungi and result in differences in the utilization intensity of different carbon sources. Therefore, although plant life form has an important influence on its rhizosphere soil fungal community, environmental conditions such as water and salt stress were more important than plant life forms in extreme environments.

### 4.3. Relationships between Carbon Metabolic Characteristics of Rhizosphere Fungal Community and Soil Properties among Different Plant Life Forms

In the oasis of arid regions, the main soil properties affecting the rhizosphere fungal metabolic characteristics were different among the different plant life forms (Table 2). The RDA results showed that the carbon to nitrogen ratio and available potassium were the main factors influencing the metabolic characteristics of the *P. euphratica* rhizosphere fungi, which is, to some extent, consistent with previous studies [61,62]. Soil salinity was the dominant factor in the variations in the rhizosphere fungal community structure of *P. euphratica*, which may further affect their carbon metabolism. The soluble sugar content in the body of *P. euphratica* increased in salt stress, which would not only reduce the demand of the rhizosphere fungal community for carbon sources in the soil but also increase the retained carbon in the soil, leading to an imbalance in the carbon to nitrogen ratio. This change may further reduce the metabolic activity of rhizosphere fungi [63]. In addition, the higher canopy of arbors requires stronger root pressure for the upward transport of water and nutrients, which may induce plants to change the osmotic pressure based on the salt content in the soil. This change may affect the water balance inside and outside of the cells of rhizosphere fungi and further influence the metabolism of rhizosphere fungi.

For *T. ramosissima*, a shrub plant with better salt tolerance [64], the main soil factor explaining the metabolic characteristics of its rhizosphere fungi is soil organic carbon. Compared with *P. euphratica*, *T. ramosissima* has a smaller canopy and leaves and relatively less apoplastic materials, so it has less input and supplementation to soil exogenous organic matter and is mostly dependent on soil background organic carbon. In addition, shrub plants in arid oases always showed a features with high root–crown ratio and deep roots [65], which may also increase the dependence of fungi on soil organic matters.

The main factor influencing the metabolic characteristics of the rhizosphere fungi of *S. alopecuroides* is the soil particle composition. The root systems of individually grown *S. alopecuroides* adopt a widespread growth strategy with maximum extension in all directions [66]. The rhizosphere soil of *S. alopecuroides* primarily consists of fine particles, predominantly silt and clay (Table 1). The variations in the clay content are related to the soil’s compactness and may further impact the root system’s expansion, additionally, a higher proportion of fine particles would decrease the soil’s air porosity. Therefore, soil clay content may indirectly influence the metabolism and growth of fungi within the root zone. A higher proportion of clay particle content may suppress the metabolic intensity of the rhizosphere fungi associated with *S. alopecuroides* [67].

## 5. Conclusions

The physicochemical properties of rhizosphere soils were significantly different among the different plant life forms in arid oases. In addition, the main soil properties affecting the variations in the metabolic characteristics of the rhizosphere fungal community were different among the different plant life forms. The dominant variables of the metabolism variations in the rhizosphere fungal community of *P. euphratica* were the carbon to nitrogen ratio (23%) and the available potassium (17.4%), while the main explanatory variables were soil organic carbon (23.1%), pH (8.6%), and total nitrogen (8.2%) for *T. ramosissima*, and the main explanatory variables were soil clay content (36.6%) and soil organic carbon (12.6%) for *S. alopecuroides*. These results suggested that the metabolic characteristics of rhizosphere fungal communities in arid oases were more affected by the soil environment than the plant species. Therefore, the improvement of the oasis soil environment should be emphasized in the management and conservation of oases in arid zones.

In this study, we used the Biolog-FF Microplate method to reflect the diversity of the fungal community, which may be insufficient to reflect the soil carbon sources actually and fungal diversity as it just includes 95 kinds of carbons, so our results may have some limitations. Together with other microbiological research methods, such as gene sequencing or microbial metabolomics, future studies might need to further explore the composition and metabolism of the rhizosphere fungal communities of different kinds of plants, which would provide new knowledge on the relationships among plants, fungal communities, and soil properties and further guide the vegetation protection and restoration in arid regions.

## Figures and Tables

**Table 1:** Physicochemical properties of rhizosphere soils among different plants.

Plant	*P. euphratica*	*T. ramosissima*	*S. alopecuroides*	Plant	*P. euphratica*	*T. ramosissima*	*S. alopecuroides*
WC (%)	13.50 ± 1.64 a	9.10 ± 0.88 b	14.01 ± 1.05 a	SOC (g/kg)	9.56 ± 1.37 a	5.90 ± 0.59 b	7.1 ± 0.59 ab
pH	8.22 ± 0.04 b	8.50 ± 0.07 a	8.47 ± 0.08 a	P (g/kg)	0.50 ± 0.01 b	0.58 ± 0.02 a	0.52 ± 0.02 b
EC (ms/cm)	0.49 ± 0.17	1.23 ± 0.60	1.03 ± 0.38	K (g/kg)	40.90 ± 1.35	43.08 ± 0.90	43.84 ± 1.87
TC (%)	2.09 ± 0.15 a	1.69 ± 0.06 b	1.96 ± 0.08 ab	Ca (g/kg)	38.04 ± 0.74 b	41.03 ± 0.64 ab	43.76 ± 2.03 a
TN (%)	0.08 ± 0.01 a	0.05 ± 0.01 b	0.06 ± 0.01 b	Na (g/kg)	10.22 ± 0.35 b	12.13 ± 0.38 a	10.84 ± 0.60 ab
C/N	29.06 ± 1.59 b	35.60 ± 1.66 a	34.19 ± 1.57 a	AN (mg/kg)	1.06 ± 0.14 a	0.61 ± 0.14 b	0.82 ± 0.10 ab
AP (mg/kg)	1.72 ± 0.18	1.91 ± 0.52	3.23 ± 2.43	AK (mg/kg)	380.38 ± 45.56	437.67 ± 168.86	372.87 ± 38.66
Sand (%)	41.39 ± 5.73 a	39.84 ± 5.83 ab	26.50 ± 3.48 b	Silt (%)	55.91 ± 5.41 b	57.70 ± 5.38 ab	71.08 ± 3.17 a
Clay (%)	2.70 ± 0.35	2.46 ± 0.51	2.42 ± 0.37				

Note: different lowercase letters in the same row indicate significant differences at 0.05 level.

**Table 2:** The contributions of soil properties on the variations in the carbon metabolic characteristics of the rhizosphere fungal community of the different plants.

Plant	Soil Properties and Contributions	Model R^2^	*p* Value
*P. euphratica*	C/N (23%), AK (17.4%)	40.4%	<0.01
*T. ramosissima*	SOC (23.1%), pH (8.6%), TN (8.2%)	39.9%	<0.05
*S. alopecuroides*	Clay (36.6%), SOC (12.6%)	49.2%	<0.01

## Data Availability

The original contributions presented in the study are included in the article, further inquiries can be directed to the corresponding author.

## References

[1] Soudzilovskaia N.A., Vaessen S., Barcelo M., He J., Rahimlou S., Abarenkov K., Brundrett M.C., Gomes S.I., Merckx V., Tedersoo L. (2020). FungalRoot: Global online database of plant mycorrhizal associations. New Phytol..

[2] Pawłowska J., Okrasińska A., Kisło K., Aleksandrzak-Piekarczyk T., Szatraj K., Dolatabadi S., Muszewska A. (2019). Carbon assimilation profiles of mucoralean fungi show their metabolic versatility. Sci. Rep..

[3] Zanne A.E., Abarenkov K., Afkhami M.E., Aguilar-Trigueros C.A., Bates S., Bhatnagar J.M., Busby P.E., Christian N., Cornwell W.K., Crowther T.W. (2020). Fungal functional ecology: Bringing a trait-based approach to plant-associated fungi. Biol. Rev..

[4] Naranjo-Ortiz M.A., Gabaldón T. (2019). Fungal evolution: Major ecological adaptations and evolutionary transitions. Biol. Rev..

[5] Singh M.P. (2009). Application of Biolog FF MicroPlate for substrate utilization and metabolite profiling of closely related fungi. J. Microbiol. Methods.

[6] Semchenko M., Leff J.W., Lozano Y.M., Saar S., Davison J., Wilkinson A., Jackson B.G., Pritchard W.J., De Long J.R., Oakley S. (2018). Fungal diversity regulates plant-soil feedbacks in temperate grassland. Sci. Adv..

[7] Yang Y., Dou Y., Huang Y., An S. (2017). Links between Soil Fungal Diversity and Plant and Soil Properties on the Loess Plateau. Front. Microbiol..

[8] Li S., Zhang Z., Wang T., Yan C., Du H. (2020). Oasis Functional Stability Evaluation Based on Multiple Indicators, Northwest China. Acta Geol. Sin..

[9] Shi X., Jiang X., Liu Y., Wu Q., Zhang Y., Li X. (2024). Evaluation of the Evolution of the Ecological Security of Oases in Arid Regions and Its Driving Forces: A Case Study of Ejina Oasis in China. Sustainability.

[10] Yu J., Wang P. (2012). Relationship between Water and Vegetation in the Ejina Delta. Agric. Water Ecol..

[11] Zhu J., Yu J., Wang P., Yu Q., Eamus D. (2014). Variability in groundwater depth and composition and their impacts on vegetation succession in the lower Heihe River Basin, north-western China. Mar. Freshw. Res..

[12] Li X., Li Y., Zhang G., Wang L., Yoshikawa K. (2017). Regeneration properties of a Populus euphratica riparian forest located in the vicinity of the Ejina Oasis, Inner Mongolia, China. Landsc. Ecol. Eng..

[13] Zhao J., Shi C., Wang D., Zhu Y., Liu J., Li H., Yang X. (2023). Sand Burial, Rather than Salinity or Drought, Is the Main Stress That Limits the Germination Ability of Sophora alopecuroides L. Seed in the Desert Steppe of Yanchi, Ningxia, China. Plants.

[14] Ju M., Zhang Q., Wang R., Yan S., Li Z., Li P., Gu P. (2022). Correlation in endophytic fungi community diversity and bioactive compounds of Sophora alopecuroides. Front. Microbiol..

[15] Bing L., Wenzhi Z., Rong Y. (2008). Characteristics and spatial heterogeneity of Tamarix ramosissima Nebkhas in desert-oasis ecotones. Acta Ecol. Sin..

[16] Yusup A., Halik Ü., Abliz A., Aishan T., Keyimu M., Wei J. (2022). Population Structure and Spatial Distribution Pattern of Populus euphratica Riparian Forest Under Environmental Heterogeneity Along the Tarim River, Northwest China. Front. Plant. Sci..

[17] Zhao P., Qu J., Xu X., Yu Q., Jiang S., Zhao H. (2019). Desert vegetation distribution and species-environment relationships in an oasis-desert ecotone of northwestern China. J. Arid Land.

[18] Cao L., Nie Z., Liu M., Wang L., Wang J., Wang Q. (2021). The Ecological Relationship of Groundwater–Soil–Vegetation in the Oasis–Desert Transition Zone of the Shiyang River Basin. Water.

[19] Shi H., Shi Q., Zhou X., Imin B., Li H., Zhang W., Kahaer Y. (2021). Effect of the competition mechanism of between co-dominant species on the ecological characteristics of Populus euphratica under a water gradient in a desert oasis. Glob. Ecol. Conserv..

[20] Zhang J., Wang X.J., Wang J.P., Wang W.X. (2014). Carbon and Nitrogen Contents in Typical Plants and Soil Profiles in Yanqi Basin of Northwest China. J. Integr. Agric..

[21] Han H., Zhai Z.H., Zhang Y.Y. (2009). BIOLOG analysis for fungal communities in environmental samples. Acta Ecol. Sin..

[22] Tang Y., Guo C., Sun X., Wang J., Han X., Liu Y. (2023). Vegetation Change Characteristics and Influencing Factors of Ejina Populus Euphratica Forest from 1977 to 2020. Desert Oasis Meteorol..

[23] Caijuan P., Qiaoyun H., Wenli C. (2017). Comparison of different BIOLOG microplate technology for detecting carbon metabolism of farmland soils. J. Huazhong Agric. Univ..

[24] Albuquerque L.G.R., Fioroto A.M., Paixão T.R.L.C., Oliveira P.V. (2017). Evaluation of Multi-Mixtures of Acids for the Sample Preparation of Organic Soil Amendments for Multi-Element Determination by ICP OES. Commun. Soil Sci. Plant Anal..

[25] Wang P., Li J., Wang Y., Liu Y., Zhang Y. (2024). Assessment of soil fertility in Xinjiang oasis cotton field based on big data techniques. Big Data Res..

[26] Su Y., Yang R. (2008). Background concentrations of elements in surface soils and their changes as affected by agriculture use in the desert-oasis ecotone in the middle of Heihe River Basin, North-west China. J. Geochem. Explor..

[27] An F., Niu Z., Liu T., Su Y. (2022). Succession of soil bacterial community along a 46-year choronsequence artificial revegetation in an arid oasis-desert ecotone. Sci. Total Environ..

[28] Suttanukool P., Darunsontaya T., Jindaluang W. (2019). A Study on the Quantity/Intensity Relationships of Potassium of Sugarcane Growing Soils, Eastern Thailand. Commun. Soil Sci. Plant Anal..

[29] Lai J., Zou Y., Zhang J., Peres-Neto P.R. (2022). Generalizing hierarchical and variation partitioning in multiple regression and canonical analyses using the rdacca.hp R package. Methods Ecol. Evol..

[30] Liu Y., Yu X., Yu Y., Hu W.H., Lai J.S. (2023). Application of “rdacca.hp” R package in ecological data analysis: Case and progress. Chin. J. Plant Ecol..

[31] Hu G.B., Dong K., Dong Y., Tang L., Zheng Y., Li X.R. (2015). Effects of intercropping oe wheat and faba bean on diversity of metabolic function of rhizosphere fungal community. Acta Pedol. Sin..

[32] Pertile G., Panek J., Oszust K., Siczek A., Frąc M. (2018). Intraspecific functional and genetic diversity of Petriella setifera. PeerJ.

[33] Zhong Y., Wang W., Wang J., Wang Y., Li J., Yuan D., Fan Y., Wei X. (2019). Leaf functional traits of oasis plants in extremely arid areas and its response to soil water and salt factors. J. Beijing For. Univ..

[34] Wu G.L., Jiang S., Liu W., Zhao C., Li J. (2016). Competition between *Populus euphratica* and *Tamarix ramosissima* seedlings under simulated high groundwater availability. J. Arid Land.

[35] Lixue J., Yan L. (2008). Comparison on Architecture Characteristics of Root Systems and Leaf Traits for Three Desert Shrubs Adapted to Arid Habitat. J. Desert Res..

[36] Zhang D., Yin L., Pan B. (2002). Biological and ecological characteristics of *Tamarix* L. and its effect on the ecological environment. Sci. China Ser. D Earth Sci..

[37] Sun Z., Long X., Ma R. (2016). Water uptake by saltcedar (*Tamarix ramosissima*) in a desert riparian forest: Responses to intra-annual water table fluctuation. Hydrol. Process..

[38] Sun L., Liu W., Chen T., Liu G. (2016). Review on Mechanism of Habitat Adaptability and Resource Value of Tamarix Species. J. Desert Res..

[39] Yu T., Feng Q., Si J., Xi H., Li Z., Chen A. (2013). Hydraulic redistribution of soil water by roots of two desert riparian phreatophytes in northwest China’s extremely arid region. Plant Soil.

[40] Nippert J.B., Butler J.J., Kluitenberg G.J., Whittemore D.O., Arnold D., Spal S.E., Ward J.K. (2010). Patterns of Tamarix water use during a record drought. Oecologia.

[41] Tengfei Y. (2013). Hydraulic Redistribution of Roots and It’s Ecohydrologic Effects for Desert Riparian Plants on the Lower Heihe River. Ph.D Thesis.

[42] Feng X., Liu R., Li C., Zhang H., Slot M. (2023). Contrasting responses of two C4 desert shrubs to drought but consistent decoupling of photosynthesis and stomatal conductance at high temperature. Environ. Exp. Bot..

[43] Wang Y., Wang J., Qu M., Li J. (2022). Plant community assembly processes and key drivers in an arid inland river basin. Biodivers. Sci..

[44] Wang F., Xu Y., Yang X., Liu Y., Lv G.H., Yang S. (2018). Soil water potential determines the presence of hydraulic lift of Populus euphratica Olivier across growing seasons in an arid desert region. J. For. Sci..

[45] Duan Q., Zhu Z., Wang B., Chen M. (2022). Recent Progress on the Salt Tolerance Mechanisms and Application of Tamarisk. Int. J. Mol. Sci..

[46] Yin C.H., Feng G., Zhang F., Tian C.Y., Tang C. (2010). Enrichment of soil fertility and salinity by tamarisk in saline soils on the northern edge of the Taklamakan Desert. Agric. Water Manag..

[47] Zhou C., Gong L., Wu X., Luo Y. (2023). Nutrient resorption and its influencing factors of typical desert plants in different habitats on the northern margin of the Tarim Basin, China. J. Arid Land.

[48] Wu H., Cui H., Fu C., Li R., Qi F., Liu Z., Yang G., Xiao K., Qiao M. (2024). Unveiling the crucial role of soil microorganisms in carbon cycling: A review. Sci. Total Environ..

[49] Yusup A., Halik U., Keyimu M., Aishan T., Abliz A., Dilixiati B., Wei J. (2023). Trunk volume estimation of irregular shaped Populus euphratica riparian forest using TLS point cloud data and multivariate prediction models. For. Ecosyst..

[50] Li S., Gou W., Wang H., White J.F., Wu G., Su P. (2021). Trade-Off Relationships of Leaf Functional Traits of Lycium ruthenicum in Response to Soil Properties in the Lower Reaches of Heihe River, Northwest China. Diversity.

[51] Chen Y., Wu S., Shi Z., Yue Y., He Y. (2023). Simulation and model comparison of the photosynthesis-light response process of *Populus euphratica* under different irrigation amounts. J. Gansu Agric. Univ..

[52] Zheng C., Qiu J., Jiang C., Yue N., Wang X., Wang W. (2007). Comparison of stomatal characteristics and photosynthesis of polymorphic *Populus euphratica* leaves. Front. For. China.

[53] Zhang Y., Wang H., Cai Y., Yang Q., Lv G. (2022). Fertile Island Effect by Three Typical Woody Plants on Wetlands of Ebinur Lake, northwestern China. Front. Environ. Sci..

[54] Shi W., Gao W., Yang Z., Ma T. (2023). Soil texture and organic carbon molecular composition are the main factors affecting soil organic carbon mineralization and microbial carbon accumulation efficiency. Pratacultural Sci..

[55] Singh M., Sarkar B., Bolan N.S., Ok Y.S., Churchman G.J. (2019). Decomposition of soil organic matter as affected by clay types, pedogenic oxides and plant residue addition rates. J. Hazard. Mater..

[56] Wang W., Li J., Wang Z., Qu L. (2017). The Soil Fungi Community Metabolic Characteristics and Influence Factors in Rhizosphere Soils of Populuseuphratica. Acta Bot. Boreali-Occident. Sin..

[57] de Vries F.T., Griffiths R.I., Bailey M., Craig H., Girlanda M., Gweon H.S., Hallin S., Kaisermann A., Keith A.M., Kretzschmar M. (2018). Soil bacterial networks are less stable under drought than fungal networks. Nat. Commun..

[58] Wen T., Yu G.-H., Hong W.-D., Yuan J., Niu G.-Q., Xie P.-H., Sun F.-S., Guo L.-D., Kuzyakov Y., Shen Q.-R. (2022). Root exudate chemistry affects soil carbon mobilization via microbial community reassembly. Fundam. Res..

[59] Jingya W., Mingliang W., Fenghua Z. (2016). Soil microbial properties under typical halophytic vegetation communities in arid regions. Acta Ecol. Sin..

[60] Oren A., Steinberger Y. (2008). Catabolic profiles of soil fungal communities along a geographic climatic gradient in Israel. Soil Biol. Biochem..

[61] Yuanyuan L. (2022). Structure of Soil Microbial Community in the Rhizosphere of *Populus euphratica* in the Lower Reaches of Tarim River and Its Correlation with Soil Physicochemical Properties. Master’s Thesis.

[62] Forogh Nasab M., Moradi M., Moradi G., Taghizadeh-Mehrjardi R. (2020). Topsoil Carbon Stock and Soil Physicochemical Properties in Riparian Forests and Agricultural Lands of Southwestern Iran. Eurasian Soil Sci..

[63] Li D., Si J., Li J., Wang P., Yuan L. (2023). Physiological responses and differences of *Populus euphratica* to salt stress and drought stress. J. Desert Res..

[64] Liu J.N., Fang H., Liang Q., Dong Y., Wang C., Yan L., Ma X., Zhou R., Lang X., Gai S. (2022). Genomic analyses provide insights into the evolution and salinity adaptation of halophyte Tamarix chinensis. Gigascience.

[65] Costa T.L., Sampaio E.V.S.B., Sales M.F., Accioly L.J.O., Althoff T.D., Pareyn F.G.C., Albuquerque E.R.G.M., Menezes R.S.C. (2014). Root and shoot biomasses in the tropical dry forest of semi-arid Northeast Brazil. Plant Soil.

[66] Hao P., Li J., Cong R., Zhang N., Jing J., Huang J. (2012). Response of arid plant Sophora alopecuroides to soil heterogeneity and competitors. J. Beijing For. Univ..

[67] Wang S.K., Zuo X.A., Zhao X.Y., Li Y.Q., Zhou X., Lv P., Luo Y.Q., Yun J.Y. (2016). Responses of soil fungal community to the sandy grassland restoration in Horqin Sandy Land, northern China. Environ. Monit. Assess..

